# From defeat to non-suicidal self-injury: the roles of entrapment and interpersonal relationship patterns in adolescence

**DOI:** 10.3389/fpsyt.2026.1889066

**Published:** 2026-08-19

**Authors:** Xiaoting Zhu, Jiaqiong Jiang

**Affiliations:** Institute of Educational Sciences, Hunan University, Changsha, China

**Keywords:** adolescents, defeat, entrapment, interpersonal relationships, latent profile analysis, non-suicidal self-injury

## Abstract

**Introduction:**

Based on the integrated motivational-volitional model and the functional theory of self-injury, this study examined the association between defeat and adolescent non-suicidal self-injury (NSSI), with entrapment as a mediator and interpersonal relationship patterns as moderators.

**Methods:**

Participants were 4,515 secondary school students (Mage = 15.24 years, SD = 1.66). Latent profile analysis was used to identify interpersonal relationship patterns across father-child, mother-child, peer, and teacher-student relationships. A moderated mediation model was then tested to examine the direct and indirect associations between defeat and NSSI across the identified profiles.

**Results:**

Overall, 33.9% of adolescents reported engaging in NSSI at least once. Defeat, entrapment, interpersonal relationships, and NSSI were significantly correlated, and entrapment partially mediated the association between defeat and NSSI. Four interpersonal relationship profiles were identified: low, moderate, and high interpersonal relationship groups and a father-child and teacher-student relationship deficit group. Interpersonal relationship profiles moderated both the direct association between defeat and NSSI and the association between defeat and entrapment. The defeat–NSSI association was strongest in the low interpersonal relationship group, whereas the defeat–entrapment association was strongest in the high interpersonal relationship group.

**Discussion:**

The findings indicate that entrapment is an important psychological mechanism linking defeat to adolescent NSSI and that the strength of these pathways varies across adolescents’ interpersonal relationship configurations. Prevention efforts may benefit from addressing both defeat-related psychological processes and adolescents’ broader relational contexts.

## Introduction

Self-injury, also referred to as non-suicidal self-injury (NSSI), is defined as the deliberate destruction of one’s own body tissue without suicidal intent and in ways that are not socially sanctioned, such as cutting, scratching, hitting, burning, and stabbing (1). NSSI is an important predictor of suicidal ideation and occurs frequently during adolescence (2). A meta-analysis estimated that the lifetime prevalence of engaging in at least one form of self-harm among adolescents worldwide ranges from 17.2% to 18.0% (3). In China, studies have reported prevalence rates of adolescent self-injury ranging from 14% to 32% (4, 5). In addition, research has shown that during the COVID-19 pandemic, the prevalence of mental health problems, including depression, anxiety, self-harm, and suicide-related outcomes, increased significantly (6, 7). Given its high prevalence and substantial risk, the etiology and development of adolescent self-injury have become important topics in psychology, sociology, and psychiatry (8).

### Defeat as a risk factor for adolescent NSSI

Defeat originates from the evolutionary theory of depression (9) and was later introduced into psychological research, where it refers to a sense of failed struggle and powerlessness resulting from the severe loss or disruption of social status, rank, or identity (10). As a factor with important implications for mental and physical health, defeat has received increasing attention in recent years (11, 12). In a study examining the cognitions associated with self-injurious urges, McEvoy et al. (13) found that feelings of defeat, self-criticism, and hopelessness were particularly pronounced during episodes of self-injury urge. Similarly, Kopetz and Orehek (14) argued that individuals experiencing social defeat may be unable to attain valued goals and may therefore engage in maladaptive behaviors, such as substance use, overeating, and self-harm, as alternative means of self-regulation. Moreover, as the severity and frequency of defeat increase, the behavioral consequences may become more serious. Consistent with this view, Laye-Gindhu and Schonert-Reichl (15) reported that 64% of adolescents endorsed the statement “I seem to have experienced failure” when describing reasons for self-injurious behavior. Accordingly, the present study proposed Hypothesis 1: defeat would be a risk factor for adolescent self-injury.

### Entrapment as a mediating mechanism linking defeat to adolescent NSSI

The integrated motivational-volitional (IMV) model, which synthesizes major theories of self-injury and suicide together with a large body of empirical evidence, proposes that the development of self-injurious and suicidal behavior unfolds across three phases: the pre-motivational phase, the motivational phase, and the volitional phase (16). Within the motivational phase, entrapment is considered a key mechanism linking defeat and humiliation to self-harm and suicidal ideation (17). The construct of entrapment derives from the notion of “arrested flight” in the cry of pain model of suicide (18) and describes the experience of being defeated while perceiving no possibility of escape. Although defeat and entrapment are closely related, they refer to different components of the IMV process. Defeat primarily captures an appraisal of failed struggle, loss of status, and powerlessness, whereas entrapment captures the subsequent perception that escape from an aversive internal or external situation is blocked. Thus, defeat concerns the meaning of the stressor for the self, while entrapment concerns perceived available options for coping or escape. In a six-month longitudinal study of 185 Scottish adolescents, Del Carpio et al. (19) found that baseline entrapment significantly predicted self-harm at follow-up, with stronger predictive effects among adolescents who had experienced bereavement.

In addition, a substantial body of research has shown that entrapment is strongly associated with suicidal ideation (20, 21), and suicidal ideation is, in turn, closely related to self-injurious behavior. Research further indicates that adolescents are at elevated risk for suicide following nonfatal self-harm (22). Moreover, both cross-sectional and longitudinal studies have shown that defeat can predict depression, anxiety, and suicidal ideation indirectly through entrapment (23, 24). The distinction between defeat and entrapment is theoretically important because adolescents may experience defeat without necessarily feeling trapped. Within the IMV model, entrapment is expected to emerge when defeat is accompanied by a perceived absence of viable escape or coping routes. Thus, although defeat and entrapment are expected to show empirical overlap, they are not conceptually interchangeable: defeat reflects an appraisal of failed struggle or loss, whereas entrapment reflects the perceived impossibility of escape. Therefore, entrapment was treated as a mediator specifying the psychological transition from defeat to NSSI risk. Based on this evidence, the present study proposed Hypothesis 2: entrapment would mediate the association between defeat and adolescent self-injury. At the same time, because adolescence is marked by rapid physical development and increasing cognitive complexity, interpersonal relationships—including family, peer, and teacher-student relationships—play a particularly important role in adolescents’ emotional and behavioral adjustment (25). Therefore, the present study further examined whether interpersonal relationships would moderate this mediational process.

### Interpersonal relationship patterns and their heterogeneity in adolescent NSSI

The functional theory of self-injury (26) posits that individuals who experience more negative interpersonal events, such as rejection, exclusion, and conflict, may develop negative self-evaluations and maladaptive cognitive schemas, thereby increasing their risk of self-injury under stressful circumstances. In a study using cluster analysis to examine adolescents’ self-injurious behavior and interpersonal relationship patterns, Stattin and Latina (27) found that, compared with adolescents characterized by safe and harmonious relationships, those with more hostile and conflictual interpersonal relationships reported higher levels of self-injurious behavior, as well as greater impulsivity, anger dysregulation, and lower self-esteem. In addition, stable and supportive parent-child relationships have been identified as protective factors that may buffer the adverse effects of stress (28). Conversely, when parent-child relationships are poor, negative life events appear to have a stronger association with adolescent self-injurious behavior (29). Similarly, studies have shown that adolescents with poorer peer relationships and teacher-student relationships are at greater risk for depression, self-injury, and suicidality in adverse environments (25).

However, previous studies have typically focused on a single type of interpersonal relationship when examining the buffering effect of stress on self-injury, or have combined parent-child, peer, and teacher-student relationships using simple summation or weighted composite scores (28, 30). Because there is no clear basis for assuming a standardized weighting across these different relational domains, such approaches may obscure meaningful heterogeneity across individuals. Latent profile analysis (LPA), a person-centered approach that identifies unobserved subgroups based on observed indicators, provides a useful alternative for capturing overall interpersonal relationship patterns across multiple relational domains (31). Accordingly, the present study used LPA to examine whether distinct interpersonal relationship patterns could be identified among adolescents. We therefore proposed Hypothesis 3: adolescent interpersonal relationships would show meaningful group heterogeneity. This person-centered approach is theoretically important because adolescents are embedded in multiple interpersonal contexts at the same time. Father-child, mother-child, peer, and teacher-student relationships provide different forms of support, belonging, guidance, and evaluation. Because these relationships serve different functions, adolescents may experience support in some domains but difficulties in others. Such relational configurations may shape how adolescents interpret defeat, whether they perceive available support or escape, and whether defeat-related distress is associated with NSSI. Thus, identifying interpersonal relationship profiles provides a more integrated understanding of how adolescents’ relational contexts shape NSSI-related risk processes.

### The present study

Grounded in the integrated motivational-volitional model and the functional theory of self-injury, the present study examined a moderated mediation model linking defeat to adolescent NSSI. Specifically, we tested whether defeat was associated with NSSI directly and indirectly through entrapment. We also used latent profile analysis to identify distinct interpersonal relationship patterns across father-child, mother-child, peer, and teacher-student relationships, and examined whether these patterns moderated the direct path from defeat to NSSI and the path from defeat to entrapment. We expected these profiles to moderate the defeat–NSSI process because different relational configurations may shape adolescents’ access to support, emotion regulation resources, and perceived possibilities for escaping or resolving defeat-related stress. Accordingly, the study addressed four questions: whether defeat predicts adolescent NSSI, whether entrapment mediates this association, whether adolescents show meaningful heterogeneity in interpersonal relationship patterns, and whether such patterns shape the proposed mediation process. The unique contribution of the present study lies in integrating the IMV model with a person-centered analysis of interpersonal relationship patterns. Unlike previous studies that examined interpersonal variables separately, this study investigates how different configurations of father-child, mother-child, peer, and teacher-student relationships moderate the pathway from defeat to entrapment and NSSI. By doing so, the present study moves beyond a variable-centered view of interpersonal relationships and provides a more integrated account of adolescents’ relational ecology.

## Methods

### Participants and procedure

The data for this study were drawn from a large-scale epidemiological survey conducted in China. Participants were recruited from several middle schools in Hunan, Guangdong, Jiangxi, and Anhui using cluster sampling. All study procedures were reviewed and approved by the ethics committee of the affiliated institution. Informed consent was obtained from the participating students, their parents or legal guardians, and the participating schools prior to data collection. Data collection was conducted by well-trained psychology teachers and graduate students, with two trained administrators present in each classroom. All participants received the same standardized instructions, and head teachers assisted during the administration process. Students were asked to read the instructions carefully and complete the questionnaires independently. After data collection, participants received small gifts, and psychological feedback was provided to schools at the class level. The study protocol was approved by the ethics review board of the affiliated institution (approval number: HUNNU-20220001).

A total of 4,515 valid questionnaires were included in the final analyses. The mean age of the sample was 15.24 years (SD = 1.66). The sample comprised 2,268 boys (50.2%) and 2,247 girls (49.8%). In addition, 1,242 participants (27.5%) were only children and 3,273 (72.5%) were non-only children. Students in the final year of junior high school (Grade 9) and the final year of senior high school (Grade 12) were not included because of the demands associated with entrance examinations. The final sample therefore consisted of 1,070 Grade 7 students (23.7%), 962 Grade 8 students (21.3%), 1,368 Grade 10 students (30.3%), and 1,114 Grade 11 students (24.7%).

### Measures

#### Non-suicidal self-injury

Adolescents’ non-suicidal self-injury (NSSI) was assessed using the Deliberate Self-Harm Inventory (DSHI; 32). This unidimensional measure has demonstrated good reliability and validity among Chinese adolescents (33). The scale consists of 9 items (e.g., “sticking sharp objects into the skin”) rated on a 6-point scale ranging from 0 to 5. Higher scores indicate greater severity of self-injurious behavior, with total scores ranging from 0 to 45. Prior research has suggested that Guttman’s lambda may provide a more appropriate lower-bound estimate of reliability than Cronbach’s alpha (34). In the present study, the Guttman’s lambda coefficient for the DSHI was.85, indicating acceptable reliability (35).

#### Defeat

Defeat was measured using the Defeat Scale developed by Gilbert and Allan (10) and revised in Chinese by Tang et al. (36). The scale contains 16 items (e.g., “I feel that I have accomplished nothing”) rated on a 5-point scale from 1 (“completely inconsistent”) to 5 (“completely consistent”). It assesses individuals’ perceptions of failed struggle and loss of social standing over the past 7 days. In the present study, the Guttman’s lambda coefficient for the scale was.87.

#### Entrapment

Entrapment was assessed using the Entrapment Scale developed by Gilbert and Allan (10). The Chinese version was adapted using a back-translation procedure by two doctoral-level English specialists and 10 psychology professionals. The scale contains 16 items (e.g., “I am in a trapped situation”) rated on a 5-point scale from 1 (“completely inconsistent”) to 5 (“completely consistent”). It measures the perceived inability to escape feelings of defeat and rejection. In the present study, the Guttman’s lambda coefficient for the scale was.95.

#### Parent-child relationship

Parent-child relationship quality was assessed using the Parent-Child Intimacy Questionnaire developed by Buchanan et al. (37). Father-child and mother-child relationships were assessed separately, with 9 items for each subscale (e.g., “You are very open when talking with your father/mother”). Items were rated on a 5-point scale ranging from 1 (“completely inconsistent”) to 5 (“completely consistent”). Higher scores indicate greater intimacy with the father or mother. In the present study, the Guttman’s lambda coefficients were.86 for the father-child subscale and.85 for the mother-child subscale.

#### Peer relationship

Peer relationship quality was measured using the short form of the Friendship Quality Questionnaire developed by Parker and Asher (38). The measure includes 18 items across six dimensions (e.g., “He/she tells me that I am very capable”) rated on a 5-point scale. Higher scores indicate better peer relationship quality. In the present study, the Guttman’s lambda coefficient for the scale was.81.

#### Teacher-student relationship

Because the head teacher typically plays a particularly central role in students’ daily school life in China, teacher-student relationship quality was assessed using the teacher-student relationship subscale from the My Class questionnaire developed by Jiang (39). This subscale focuses primarily on students’ relationships with their head teachers and includes 8 items (e.g., “The head teacher is kind and approachable”). Items were rated on a 5-point scale ranging from 1 (“never”) to 5 (“always”). In the present study, the Guttman’s lambda coefficient for the scale was.92.

### Data analysis

All analyses were conducted using SPSS 26, AMOS 23, and Mplus 7. First, common method bias was examined using Harman’s single-factor test and a confirmatory factor analysis of a single-factor model. Second, descriptive statistics and bivariate correlations were computed for all study variables. Third, because the interpersonal relationship measures differed in the number of items and response formats, scores for father-child, mother-child, peer, and teacher-student relationships were standardized and transformed to T-scores prior to latent profile analysis (LPA). LPA was then conducted to identify distinct interpersonal relationship patterns among adolescents, with model selection based on fit indices including AIC, BIC, adjusted BIC, entropy, the Lo-Mendell-Rubin test, and the bootstrap likelihood ratio test. Finally, the hypothesized moderated mediation model was tested using PROCESS 3.3. Defeat was entered as the independent variable, entrapment as the mediator, adolescent non-suicidal self-injury as the dependent variable, and interpersonal relationship patterns as the moderator, with gender included as a control variable. All continuous variables were standardized prior to analysis, and simple slope analyses were conducted to probe significant interaction effects.

## Results

### Common method bias

Because all data were obtained through self-report measures, the possibility of common method bias could not be ruled out. Harman’s single-factor test was first conducted using exploratory factor analysis on all items from the six questionnaires, with an unrotated principal component solution. The results showed that 13 factors had eigenvalues greater than 1, and the first factor accounted for 20.82% of the total variance, which was below the critical threshold of 40%. In addition, following Wu and Wen (40), the three items with the highest factor loadings from each scale were parceled as indicators of the corresponding latent variables. A confirmatory factor analysis of the single-factor model conducted in AMOS showed poor model fit, χ² = 34232.84, df = 189, CFI = .39, TLI = .32, RMSEA = .20, SRMR = .15. These findings suggest that common method bias was not a serious concern in the present study.

### Descriptive statistics and correlations

**Table 1** presents the means, standard deviations, and bivariate correlations for all study variables. Because previous research has shown that NSSI is significantly associated with gender (41), gender was also included in the correlation analysis. The results indicated that NSSI, defeat, and entrapment were positively correlated with one another, whereas father-child, mother-child, peer, and teacher-student relationships were negatively correlated with NSSI, defeat, and entrapment and positively correlated with one another. Gender was positively correlated with NSSI, entrapment, mother-child relationship quality, and peer relationship quality. In addition, 33.9% of the adolescents in the present sample reported engaging in NSSI at least once.

**Table 1:** Means, standard deviations, and correlations among study variables.

Variable	1	2	3	4	5	6	7	8
1. NSSI	1							
2. Defeat	0.34^**^	1						
3. Entrapment	0.34^**^	0.71^**^	1					
4. Father-child relationship	-0.15^**^	-0.30^**^	-0.38^**^	1				
5. Mother-child relationship	-0.18^**^	-0.20^**^	-0.36^**^	0.78^**^	1			
6. Peer relationship	-0.05^**^	-0.19^**^	-0.20^**^	0.24^**^	0.26^**^	1		
7. Teacher-student relationship	-0.11^**^	-0.20^**^	-0.22^**^	0.25^**^	0.22^**^	0.21^**^	1	
8. Gender	0.05^**^	0.01	0.09^**^	-0.01	0.05^**^	0.13^**^	-0.01	1
*M* ± *SD*	2.72 ± 6.47	35.10 ± 10.94	37.13 ± 16.82	28.54 ± 8.42	30.21 ± 8.04	63.54 ± 13.12	30.97 ± 8.42	—

1 = NSSI; 2 = defeat; 3 = entrapment; 4 = father-child relationship; 5 = mother-child relationship; 6 = peer relationship; 7 = teacher-student relationship; 8 = gender. Gender was coded as 1 = male and 2 = female. **p < .01.

### Adolescent interpersonal relationship patterns: latent profile analysis

Because the interpersonal relationship measures differed in item number and response format, scores for each scale were standardized and transformed into T-scores with a mean of 50 and a standard deviation of 10. Latent profile analysis was then conducted to identify adolescents’ interpersonal relationship patterns, and the fit indices for the competing models are shown in **Table 2**. The 4-, 5-, and 6-profile solutions all showed entropy values above.80, indicating acceptable classification accuracy (42). Although the five-profile solution had lower AIC, BIC, and adjusted BIC values than the four-profile solution, the four-profile solution showed slightly higher entropy and offered a more parsimonious and interpretable classification. In addition, the LMR and BLRT results for the six-profile solution were not significant. Therefore, considering model fit, classification accuracy, parsimony, and interpretability together, the four-profile solution was retained as the final model.

**Table 2:** Fit indices for the latent profile analysis models.

Class	AIC	BIC	aBIC	Entropy	LMR	BLRT	Class probability
1	134433.7	134485.1	134459.6				
2	131746.4	131829.8	131788.5	0.689	2697.328***	2634.710***	0.55, 0.45
3	130254.8	130370.3	130313.1	0.782	1501.614***	1466.755***	0.14, 0.34, 0.52
4	129770.7	129918.2	129845.2	0.843	494.099**	482.629**	0.15, 0.49, 0.33, 0.03
5	128968.3	129147.9	129059	0.835	812.365*	793.506**	0.12, 0.04, 0.36, 0.11, 0.37
6	128383.7	128595.4	128490.5	0.847	594.652	580.847	0.14, 0.05, 0.32, 0.35, 0.04, 0.10

AIC, Akaike information criterion; BIC, Bayesian information criterion; aBIC, adjusted Bayesian information criterion; LMR, Lo-Mendell-Rubin likelihood ratio test; BLRT, bootstrap likelihood ratio test. *p < .05, **p < .01, ***p < .001 .

To further clarify the characteristics of the four latent profiles, **Table 3** presents comparisons across the four groups on each interpersonal relationship dimension. One-way ANOVAs showed significant between-group differences for father-child, mother-child, peer, and teacher-student relationships, as well as for the overall composite score. Specifically, father-child relationship scores followed the pattern Group 4 < Group 1 < Group 2 < Group 3; mother-child relationship scores followed the pattern Group 1 < Group 2 < Group 4 < Group 3; peer relationship scores were lowest in Group 1, followed by Group 2, whereas Groups 3 and 4 showed relatively higher scores; teacher-student relationship scores followed the pattern Groups 1 and 4 < Group 2 < Group 3. For the overall composite score, the ordering was Group 1 < Group 4 < Group 2 < Group 3. Based on these patterns, the four profiles were labeled as follows: a low interpersonal relationship group (n = 661, 15%), a moderate interpersonal relationship group (n = 2,232, 49%), a high interpersonal relationship group (n = 1,483, 33%), and a father-child and teacher-student relationship deficit group (n = 139, 3%).

**Table 3:** Comparisons of the latent profiles across interpersonal relationship dimensions.

Profile	Father-child relationship	Mother-child relationship	Peer relationship	Teacher-student relationship	Composite score
Total	50.00 ± 10.00	50.00 ± 10.00	50.00 ± 10.00	50.00 ± 10.00	50.00 ± 7.03
Group 1	35.15 ± 5.01	33.56 ± 5.58	46.04 ± 11.21	46.38 ± 11.68	40.28 ± 5.09
Group 2	48.59 ± 4.40	47.98 ± 4.97	48.31 ± 9.00	48.73 ± 9.39	48.40 ± 4.02
Group 3	60.37 ± 5.00	59.76 ± 4.95	54.09 ± 9.57	53.75 ± 8.88	56.99 ± 4.20
Group 4	32.57 ± 4.28	56.47 ± 5.82	52.29 ± 8.45	47.57 ± 10.00	47.23 ± 4.05
*F*	5326.92***	4314.14***	122.22***	154.96***	2613.29***
Multiple comparisons	4 < 1 < 2 < 3	1 < 2 < 4 < 3	1 < 2 < 4, 3	1 4 < 2 < 3	1 < 4 < 2 < 3

***p < .001.

### The association between defeat and adolescent NSSI: a moderated mediation analysis

Following the procedure for testing moderated mediation proposed by Wen and Ye (43), the present study examined the association between defeat and adolescent NSSI, the mediating role of entrapment, and the moderating role of interpersonal relationship patterns, while controlling for gender. All continuous variables were standardized prior to analysis, and all analyses were conducted using Hayes’s PROCESS macro (Version 3.3) in SPSS.

As shown in **Table 4**, in Equation 1, gender, defeat, interpersonal relationship patterns, and their interaction terms significantly predicted adolescent NSSI, indicating that interpersonal relationship patterns moderated the association between defeat and adolescent NSSI. In Equation 2, gender, defeat, interpersonal relationship patterns, and the interaction terms significantly predicted entrapment. In Equation 3, gender, defeat, entrapment, interpersonal relationship patterns, and the interaction terms all significantly predicted adolescent NSSI. These findings indicate that defeat, entrapment, adolescent NSSI, and interpersonal relationship patterns constituted a moderated mediation model. Specifically, entrapment partially mediated the association between defeat and adolescent NSSI, and interpersonal relationship patterns moderated both the direct path and the first stage of the mediation process. In addition, **Table 5** presents the indirect effects, direct effects, and the proportion of the total effect accounted for by mediation across the different interpersonal relationship profiles. The results showed that the indirect effects were largest in Groups 3 and 4, whereas the direct effect in Group 4 was not significant, despite this group showing the highest proportion of the mediated effect.

**Table 4:** Moderated mediation analysis predicting adolescent non-suicidal self-injury.

Variable	Equation 1 (*Y* = NSSI)					Equation 2 (*M* = Entrapment)					Equation 3 (*Y* = NSSI)				
	β	*SE*	*t*	95% CI		β	*SE*	*t*	95% CI			β	*SE*	*t*	95% CI
Gender	0.90	0.03	3.29^**^	[0.36, 0.14]		0.16	0.02	7.82^***^	[0.12, 0.20]			0.06	0.03	2.19^*^	[0.01, 0.11]
X	0.41	0.03	13.71^***^	[0.35, 0.47]		0.60	0.02	27.02^***^	[0.56, 0.64]			0.30	0.03	9.25^***^	[0.23, 0.36]
W1	-0.26	0.04	-6.01^***^	[-0.35, -0.18]		-0.24	0.03	-7.33^***^	[-0.03, -0.17]			-0.22	0.04	-4.99^***^	[-0.30, -0.13]
W2	-0.28	0.05	-5.87^***^	[-0.37, -0.19]		-0.52	0.04	-14.74^***^	[-0.59, -0.45]			-0.18	0.05	-3.73^**^	[-0.27, -0.09]
W3	-0.33	0.09	-3.68^**^	[-0.21, -0.07]		-0.16	0.07	-2.36^*^	[-0.29, -0.03]			-0.30	0.09	-3.38^**^	[-0.47, -0.13]
X*W1	-0.14	0.04	-3.84^**^	[-0.21, -0.07]		0.05	0.03	1.98^*^	[0.01, 0.11]			-0.15	0.04	-4.16^***^	[-0.22, -0.08]
X*W2	-0.13	0.04	-3.14^**^	[-0.21, -0.05]		0.08	0.03	2.71^**^	[0.02, 0.14]			-0.15	0.04	-3.56^**^	[-0.23, -0.07]
X*W3	-0.20	0.09	-2.12^*^	[-0.38, -0.01]		0.07	0.07	1.00	[-0.07, 0.20]			-0.21	0.09	-2.28^*^	[-0.39, -0.03]
M												0.19	0.02	9.53^***^	[0.15, 0.23]
*R^2^*	0.13				0.53					0.15					
*F*	6.23^***^				627.31^***^					83.70^***^					

Analyses were conducted using PROCESS 3.3 in SPSS. X = defeat; M = entrapment; Y = adolescent non-suicidal self-injury (NSSI). W1, W2, and W3 represent dummy-coded interpersonal relationship profiles, with the reference group omitted from the table. Bold values indicate statistically significant effects. *p < .05, **p < .01, ***p < .001.

**Table 5:** Indirect and direct effects across interpersonal relationship profiles.

Moderator	Group	Indirect effect	Bootstrap SE	95% CI	Direct effect	Bootstrap SE	95% CI	Proportion mediated
Interpersonal relationship pattern	Group1	0.11	0.02	[0.08, 0.15]	0.30	0.03	[0.23, 0.36]	27%
	Group2	0.12	0.02	[0.09, 0.16]	0.14	0.02	[0.10, 0.19]	46%
	Group3	0.13	0.02	[0.09, 0.17]	0.15	0.03	[0.09 0.21]	46%
	Group4	0.13	0.02	[0.09, 0.18]	0.09	0.09	[-0.09, 0.26]	59%

Bold values indicate statistically significant effects.

**Figure 1:**
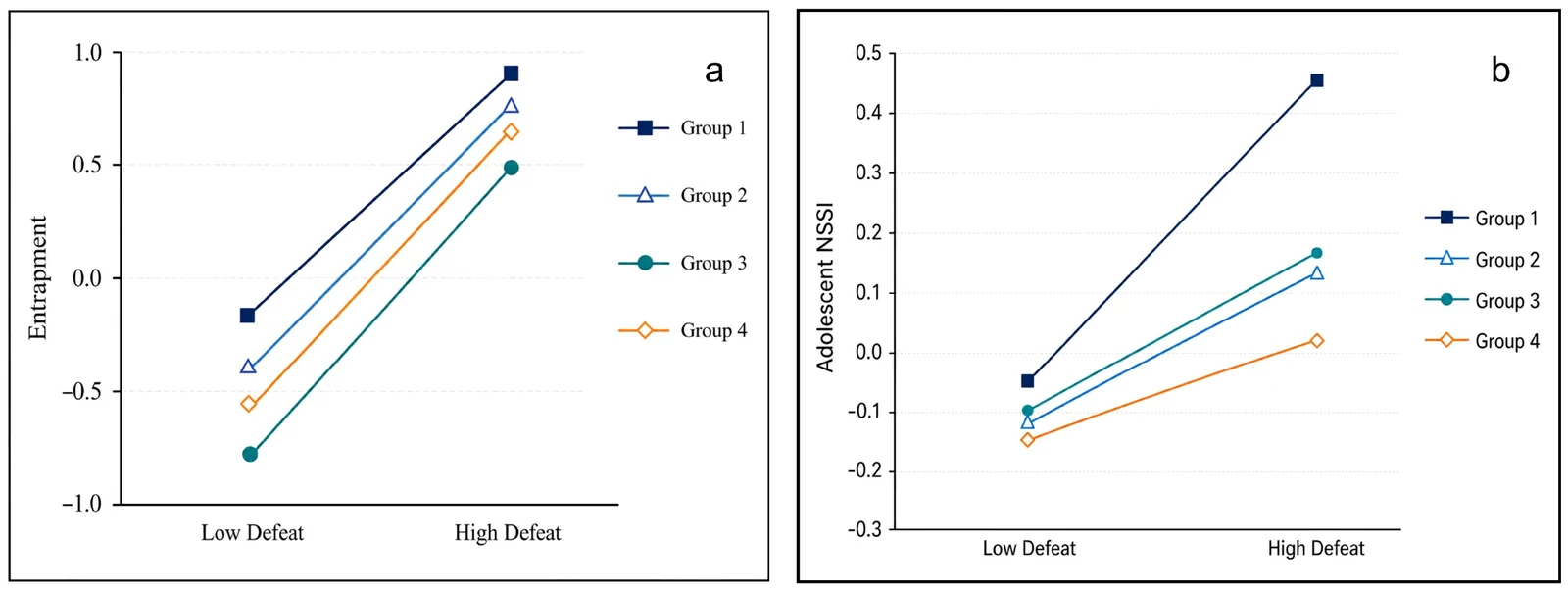
Interpersonal relationship patterns moderate the associations of defeat with entrapment and adolescent NSSI. **(a)** shows the moderating effect of interpersonal relationship patterns on the association between defeat and entrapment. **(b)** shows the moderating effect of interpersonal relationship patterns on the association between defeat and adolescent NSSI. Group 1 = low interpersonal relationship group; Group 2 = moderate interpersonal relationship group; Group 3 = high interpersonal relationship group; Group 4 = father-child and teacher-student relationship deficit group. Low defeat and high defeat represent low and high levels of defeat, respectively.

To further interpret the moderating effects, simple slope analyses were conducted to examine the role of interpersonal relationship patterns in both the direct path and the first stage of the mediation model. As shown in **Figure 1**, defeat significantly predicted entrapment across all four interpersonal relationship profiles (β_Group 1_ = 0.59, *t* = 27.02, *p* <.001; β_Group 2_ = 0.65, *t* = 41.44, *p* <.001; β_Group 3_ = 0.68, *t* = 31.80, *p* <.001; β_Group 4_ = 0.67, *t* = 10.26, *p* <.001). The slope was steepest in Group 3, the high interpersonal relationship group. Moreover, across levels of defeat, adolescents in Group 3 reported lower levels of entrapment than those in the other three groups.

In addition, defeat significantly predicted adolescent NSSI in the first three interpersonal relationship profiles (β_Group 1_ = 0.29, *t* = 9.25, *p* <.001; β_Group 2_ = 0.14, *t* = 5.83, *p* <.001; β_Group 3_ = 0.15, *t* = 4.68, *p* <.001), but not in Group 4 (β_Group 4_ = 0.09, *t* = 0.98, *p* = .33). Among the four groups, the slope was steepest in Group 1, the low interpersonal relationship group, which also showed the highest level of adolescent NSSI.

## Discussion

The present study examined whether entrapment mediated the association between defeat and adolescent NSSI and whether this process differed across interpersonal relationship patterns. Consistent with the integrated motivational-volitional model, defeat was associated with NSSI both directly and indirectly through entrapment. More importantly, these pathways varied across adolescents’ interpersonal relationship profiles. By integrating the IMV model with a person-centered perspective, this study extends previous research in two ways. First, it highlights entrapment as a key psychological process through which defeat-related distress may be linked to NSSI. Second, it shows that interpersonal relationships may shape this process not only as separate protective or risk factors, but also as broader relational configurations. Because adolescents may experience supportive relationships in some domains while facing difficulties in others, examining relational profiles can reveal patterns of vulnerability and protection that may be obscured when father-child, mother-child, peer, and teacher-student relationships are considered separately.

### Defeat and entrapment as key psychological processes underlying adolescent NSSI

The present findings suggest that defeat may be an important psychological risk factor for adolescent NSSI, both directly and indirectly through entrapment. On the one hand, defeat was directly associated with higher levels of NSSI, indicating that adolescents who perceive themselves as having failed, lost status, or become powerless may be more likely to engage in self-injury. This finding is consistent with the experiential avoidance model, which emphasizes that self-harm can serve as a maladaptive strategy for escaping or regulating aversive internal states (44). When adolescents experience defeat, the resulting sense of frustration, inferiority, and helplessness may become difficult to tolerate, thereby increasing the likelihood of self-injurious behavior as a means of coping. This interpretation is also in line with previous work showing that defeat is associated with self-handicapping and self-harm-related cognitions (45).

On the other hand, the results further showed that entrapment partially mediated the association between defeat and adolescent NSSI, which provides more specific insight into how defeat may translate into self-injurious behavior. The mediating role of entrapment should be understood in terms of this conceptual boundary. Defeat reflects a self-referential appraisal that one has failed, lost status, or been overpowered. Entrapment, by contrast, reflects a motivational state in which the adolescent perceives no acceptable way to escape from the consequences of that defeat. The high correlation observed in the present study suggests that these experiences often co-occur; however, co-occurrence does not mean that they are interchangeable. In the IMV framework, defeat is theorized to increase risk when it progresses into entrapment, because the perceived absence of escape may make self-injury more likely to function as an immediate means of relief.

According to the integrated motivational-volitional model, defeat alone may not be sufficient to produce severe self-harm risk; rather, its effects become especially potent when adolescents also perceive themselves as trapped and unable to escape from adverse circumstances (16). In other words, it may be the combination of “being defeated” and “seeing no way out” that heightens vulnerability to NSSI. For adolescents facing social setbacks, interpersonal stress, or status-related threats, the absence of effective coping resources may intensify feelings of entrapment, which in turn may increase the appeal of self-injury as an immediate, though maladaptive, means of relief. Thus, the present findings not only support the relevance of defeat and entrapment in understanding adolescent NSSI, but also highlight entrapment as a key explanatory process linking adverse self-referential experiences to self-injurious behavior. From a practical perspective, these results suggest that prevention and intervention efforts may benefit from paying closer attention to adolescents’ subjective experiences of defeat and entrapment, rather than focusing solely on overt behavioral risk markers.

### Interpersonal relationship patterns as contextual buffers and vulnerability factors

The present study further showed that interpersonal relationship patterns played an important moderating role in the association between defeat, entrapment, and adolescent NSSI. According to the functional theory of self-injury, interpersonal relationships may shape adolescents’ responses to stress by either buffering or amplifying the effects of adverse experiences (26). Supportive relationships may provide emotional security, coping resources, and opportunities for co-regulation, thereby reducing the likelihood that stress will escalate into self-injurious behavior. In contrast, poor interpersonal relationships may heighten adolescents’ vulnerability to NSSI by increasing negative affect, maladaptive cognitions, and stress sensitivity (46). Against this background, the present study moved beyond prior research that focused on a single relational domain or combined multiple relationships using simple summed or weighted scores. Instead, by examining father-child, mother-child, peer, and teacher-student relationships simultaneously within key microsystems of adolescent development (47), the study identified four distinct interpersonal relationship profiles, highlighting meaningful heterogeneity in adolescents’ relational environments.

The identified profiles reflect adolescents’ broader relational ecology rather than isolated relationship qualities. A uniformly low relationship profile may indicate limited support across multiple contexts, whereas a uniformly high profile may reflect a more coherent network of relational resources. An imbalanced profile, such as the father-child and teacher-student relationship deficit group, suggests that vulnerability may be concentrated in relationships involving authority, evaluation, and external expectations. These relational contexts may shape how adolescents interpret defeat and whether they perceive available support or escape. The high interpersonal relationship group showed the lowest overall level of entrapment but the strongest association between defeat and entrapment. This pattern suggests that supportive relationships may reduce chronic feelings of being trapped, but may not fully buffer adolescents from acute experiences of defeat. By contrast, the low interpersonal relationship group showed the highest level of NSSI and the strongest direct association between defeat and NSSI, suggesting that when relational resources are broadly lacking, defeat-related distress may be more readily associated with self-injurious behavior.

The father-child and teacher-student relationship deficit group may also be theoretically informative. Because this profile included only 3% of the sample, however, the specific pattern observed in this group should be interpreted cautiously and requires replication. Despite its relatively better mother-child and peer relationship scores, the deficits in father-child and teacher-student relationships may still reflect a meaningful vulnerability pattern. One possible interpretation is that these adolescents experience tension specifically in relationships characterized by authority, evaluation, or control. Compared with mothers, fathers may more often be perceived as stricter or more hierarchical in some family contexts, which may increase the likelihood of father-child conflict (48). Similarly, difficulties in teacher-student relationships may reflect discomfort with authority figures or negative responses to externally imposed expectations. From this perspective, it is notable that the direct effect of defeat on NSSI was not significant in this group, whereas the mediated effect through entrapment remained relatively strong. This pattern may suggest that, for adolescents with this particular relational configuration, defeat may be more likely to operate through internalized feelings of entrapment rather than through a direct pathway to self-injury. The moderated mediation effects should be interpreted as indicating relative vulnerability rather than large clinical differences, with the strongest practical implication being that adolescents with high defeat and poor interpersonal relationships may warrant closer attention in NSSI risk screening. Overall, these findings underscore that interpersonal relationships are not merely background correlates of adolescent NSSI; rather, they constitute an important contextual system that shapes how adolescents experience defeat and whether such experiences are translated into entrapment and self-injurious behavior.

### Practical and clinical implications

The present findings have several practical and clinical implications for the prevention and intervention of adolescent NSSI. First, the results suggest that defeat and entrapment may serve as important psychological warning signs in the early identification of adolescents at risk for NSSI. In school mental health screening and clinical assessment, practitioners may benefit from paying closer attention not only to overt self-injurious behaviors, but also to adolescents’ subjective experiences of failed struggle, helplessness, and feeling trapped, as these internal experiences may precede or intensify self-injury risk. Second, the identification of distinct interpersonal relationship profiles highlights the importance of adopting a person-centered perspective in intervention planning. However, these profile differences should be interpreted as indicators of relative vulnerability rather than as clinical cutoffs. In practice, adolescents who report high defeat and entrapment together with broadly poor interpersonal relationships may warrant closer attention in school mental health screening and preventive intervention. Adolescents with generally poor interpersonal relationships may require broader support across family, peer, and school contexts, whereas those showing specific vulnerabilities in father-child and teacher-student relationships may benefit from more targeted relational interventions focused on authority-related conflict, communication, and emotional support. However, implications for the father-child and teacher-student relationship deficit group should be considered preliminary given the small size of this profile. Third, the moderating role of interpersonal relationship patterns suggests that strengthening supportive relationships may reduce the extent to which defeat develops into entrapment and, ultimately, into NSSI. Accordingly, preventive and clinical efforts may benefit from combining individual-level interventions, such as coping skills training, emotion regulation, and cognitive restructuring, with relational interventions aimed at improving parent-child communication, teacher-student support, and peer connectedness. Overall, the present study suggests that effective intervention for adolescent NSSI may require not only reducing intrapersonal distress, but also improving the relational contexts in which that distress unfolds.

### Limitations and future directions

Several limitations of the present study should be noted. First, although the study was based on a relatively large sample, the cross-sectional design precludes conclusions about temporal or causal relations among defeat, entrapment, and adolescent NSSI. Future longitudinal studies are needed to examine whether the proposed sequence from defeat to entrapment and then to NSSI unfolds over time. Given the strong correlation between defeat and entrapment, future research should also further test their discriminant validity. Second, although latent profile analysis helped identify distinct interpersonal relationship patterns, this person-centered approach made it difficult to isolate the unique role of each relationship domain. Future research may combine person-centered and variable-centered approaches to clarify how father-child, mother-child, peer, and teacher-student relationships differentially shape adolescents’ vulnerability to NSSI. Third, the functional theory of self-injury suggests that NSSI may also serve interpersonal functions, such as eliciting attention, support, or reductions in interpersonal conflict. Therefore, the association between interpersonal relationships and adolescent NSSI may be reciprocal rather than unidirectional. Future research should further examine these dynamic bidirectional processes. Finally, given the large sample size, statistically significant associations may be modest in practical magnitude. Further work should examine the predictive utility, clinical thresholds, and incremental validity of these profiles for identifying adolescents at risk of NSSI.

## Conclusion

The present study advances understanding of adolescent NSSI by showing that defeat is associated with NSSI both directly and indirectly through entrapment, and that these processes are shaped by adolescents’ interpersonal relationship patterns. By integrating the integrated motivational-volitional model with a person-centered approach to interpersonal relationships, this study highlights both the psychological and relational contexts in which adolescent NSSI develops. These findings suggest that adolescent NSSI should be understood not only as an individual response to internal distress, but also as a behavior embedded in broader interpersonal environments. Accordingly, efforts to prevent and intervene in adolescent NSSI may benefit from addressing both intrapersonal risk processes, such as defeat and entrapment, and the relational contexts that may either buffer or exacerbate these risks.

## Data Availability

The raw data supporting the conclusions of this article will be made available by the authors, without undue reservation.
